# Abdominal fat and muscle distributions in different stages of colorectal cancer

**DOI:** 10.1186/s12885-023-10736-2

**Published:** 2023-03-28

**Authors:** Jun Han, Xinyang Liu, Min Tang, Fan Yang, Zuoyou Ding, Guohao Wu

**Affiliations:** 1 Department of General Surgery, Zhongshan Hospital, Fudan University, 180 Fenglin Road, Shanghai, 200032 China; 2 Shanghai Clinical Nutrition Research Center, Shanghai, China; 3 Endoscopy Center and Endoscopy Research Institute, Zhongshan Hospital, Fudan University, Shanghai, China; 4 Department of Radiology, Zhongshan Hospital, Fudan University, Shanghai, China

**Keywords:** Colorectal cancer, Cancer cachexia, Colorectal polyp, Subcutaneous adipose tissue, Visceral adipose tissue

## Abstract

**Background:**

The purpose of this study is to explore the difference of abdominal fat and muscle composition, especially subcutaneous and visceral adipose tissue, in different stages of colorectal cancer (CRC).

**Materials and methods:**

Patients were divided into 4 groups: healthy controls (patients without colorectal polyp), polyp group (patients with colorectal polyp), cancer group (CRC patients without cachexia), and cachexia group (CRC patients with cachexia). Skeletal muscle (SM), subcutaneous adipose tissue (SAT), visceral adipose tissue (VAT), and intermuscular adipose tissue (IMAT) were assessed at the third lumbar level on computed tomography images obtained within 30 days before colonoscopy or surgery. One-way ANOVA and linear regression were used to analyze the difference of abdominal fat and muscle composition in different stages of CRC.

**Results:**

A total of 1513 patients were divided into healthy controls, polyp group, cancer group, and cachexia group, respectively. In the development of CRC from normal mucosa to polyp and cancer, the VAT area of the polyp group was significantly higher than that of the healthy controls both in male (156.32 ± 69.71 cm^2^ vs. 141.97 ± 79.40 cm^2^, *P* = 0.014) and female patients (108.69 ± 53.95 cm^2^ vs. 96.28 ± 46.70 cm^2^, *P* = 0.044). However, no significant differences were observed of SAT area between polyp group and healthy controls in both sexes. SAT area decreased significantly in the male cancer group compared with the polyp group (111.16 ± 46.98 cm^2^ vs. 126.40 ± 43.52 cm^2^, *P* = 0.001), while no such change was observed in female patients. When compared with healthy controls, the SM, IMAT, SAT, and VAT areas of cachexia group was significantly decreased by 9.25 cm^2^ (95% CI: 5.39–13.11 cm^2^, *P* < 0.001), 1.93 cm^2^ (95% CI: 0.54–3.32 cm^2^, *P* = 0.001), 28.84 cm^2^ (95% CI: 17.84–39.83 cm^2^, *P* < 0.001), and 31.31 cm^2^ (95% CI: 18.12–44.51 cm^2^, *P* < 0.001) after adjusting for age and gender.

**Conclusion:**

Abdominal fat and muscle composition, especially SAT and VAT, was differently distributed in different stages of CRC. It is necessary to pay attention to the different roles of subcutaneous and visceral adipose tissue in the development of CRC.

**Supplementary Information:**

The online version contains supplementary material available at 10.1186/s12885-023-10736-2.

## Introduction

Colorectal cancer (CRC) remains a major health burden with high mortality throughout the world. Globally, there are 1.8 million cases and 880,792 deaths from CRC in 2018 [1]. With the changes in lifestyle such as the lack of physical activity, and the increasing prevalence of obesity in recent decades, the incidence of CRC in China has been raised [2]. It is well known that colorectal polyp is a key step in CRC development. Different polyp subtypes lead to cancer development through distinct neoplasia pathways, in which the adenoma-carcinoma pathway contributes up to 60–70% of all CRC [3]. A number of epidemiological studies have reported an association between the risk for CRC and obesity [4, 5]. Visceral obesity was reported as a risk factor for colorectal adenoma [6]. However, whether adipose tissue is increasing from adenoma to carcinoma is still unclear.

Human white adipose tissue is a prominent energy reservoir and can be categorized into subcutaneous adipose tissue (SAT) and visceral adipose tissue (VAT) [7]. Recent studies have shown that the metabolic characteristics and embryonic origin of SAT and VAT are different [7–9]. Increased VAT is a risk factor for various tumors, including CRC [10, 11]. VAT is also associated with a higher incidence of colorectal adenoma in a dose-dependent manner [12]. On the other hand, the increase of SAT is not associated with CRC and is even negatively associated with CRC in African Americans [13]. In addition, the prognostic value of SAT and VAT is also different in various cancers [14, 15]. Intramuscular adipose tissue (IMAT) is a measure of adipose tissue infiltration in skeletal muscle fibers [16]. IMAT highly correlates with muscle density and can lead to a higher risk of adverse health outcomes [17, 18]. However, the role of IMAT in the development of CRC is unclear.

Measurement of waist circumference and body mass index (BMI) are two conventional methods to determine the abdominal fat and muscle composition. However, such methods cannot accurately distinguish SAT and VAT. In recent years, with the application of imaging techniques such as computed tomography (CT) scans, abdominal fat and muscle composition can be accurately segmented to be SAT and VAT as well as IMAT and skeletal muscle (SM) [19, 20]. The third lumbar vertebra (L3) is a common reference point for the estimation of abdominal fat and muscle composition [21, 22]. With its power to use neural networks and convolutional layers to learn the hierarchy of features from a large amount of given data, deep learning systems can be trained to analyze abdominal fat and muscle composition [23, 24]. In a previous study, we have developed a V-Net-Based segmentation deep learning system to segment skeletal muscle and adipose tissues quickly and accurately [15]. It provides a useful method for large-scale calculation of human abdominal fat and muscle composition (SM, SAT, VAT, and IMAT).

Cancer cachexia is a common phenomenon of advanced tumors, which is mainly characterized by loss of skeletal muscle and adipose tissues [25]. A large number of studies have shown that skeletal muscle atrophy is an independent prognostic factor of cancer patients with cachexia [26–28]. However, prognostic value of adipose tissue loss in patients with cancer cachexia is still controversial [15, 29, 30]. Given CRC often occurs in obese patients, it is not clear whether CRC patients with cachexia would experience adipose tissue loss as other cancer patients do.

In this study, we compared the abdominal fat and muscle composition differences in different stages of colorectal cancer (patients with and without colorectal polyp, CRC patients with and without cachexia), so as to provide evidence for the clinical prevention and treatment of CRC.

## Materials and methods

### Patients and groups

Patients with CRC who underwent surgery in the Department of General Surgery from January 2020 to December 2020 and patients who underwent colonoscopy in Endoscopic Center during this period were selected in Zhongshan Hospital, Fudan University. Inclusion criteria: (1) patients with CRC were pathologically diagnosed as colorectal adenocarcinoma; (2) patients with colorectal polyp were detected by colonoscopy and pathologically confirmed as adenomas; (3) healthy controls were confirmed by colonoscopy and no polyp was found; (4) patients performed abdominal CT scans within 30 days before surgery or colonoscopy. The diagnostic criteria of cancer cachexia referred to the international consensus on cancer cachexia proposed in 2011 as weight loss of more than 5% in the past 6 months [25]. In this study, all patients were divided into 4 groups: healthy controls (patients without colorectal polyp), polyp group (patients with colorectal polyp), cancer group (CRC patients without cachexia), and cachexia group (CRC patients with cachexia). The patient’s age and gender were recorded in all 4 groups of patients. Cancer stages were recorded based on the American Joint Committee on Cancer stage (8th edition) groupings in CRC groups with and without cachexia. The ethics committee of Zhongshan Hospital, Fudan University approved this study.

### Adipose tissue and muscle areas determination of abdominal CT

Abdominal CT scans were performed within 30 days before colonoscopy or surgery. CT parameters for each patient were as follows: contrast-enhanced or unenhanced, 120 kVp, and 290 mA. The scanning layer was 1–5 mm thick and ranged from the xiphoid process to pubic symphysis. The areas of SAT, VAT, SM, as well as IMAT, were segmented by previously described method by our team [15]. A representative CT image marked with different parts of adipose tissue and SM was shown in Fig. S1.

### Statistical analyses

Categorical variables were described as a number with percentages and were compared using χ^2^ test. Continuous variables were described as mean with standard deviation and were compared using one-way ANOVA and linear regression. A Bonferroni correction was applied to adjust for multiple comparisons in one-way ANOVA. Univariate and multivariate linear regression were both adopted to evaluate the crude and adjusted difference among different disease statuses. Two-sided tests were used, and a *P*-value < 0.05 was considered statistically significant. All statistical analyses were carried out with Stata 14.0.

## Results

### Characteristics of enrolled patients

**Fig. 1:**
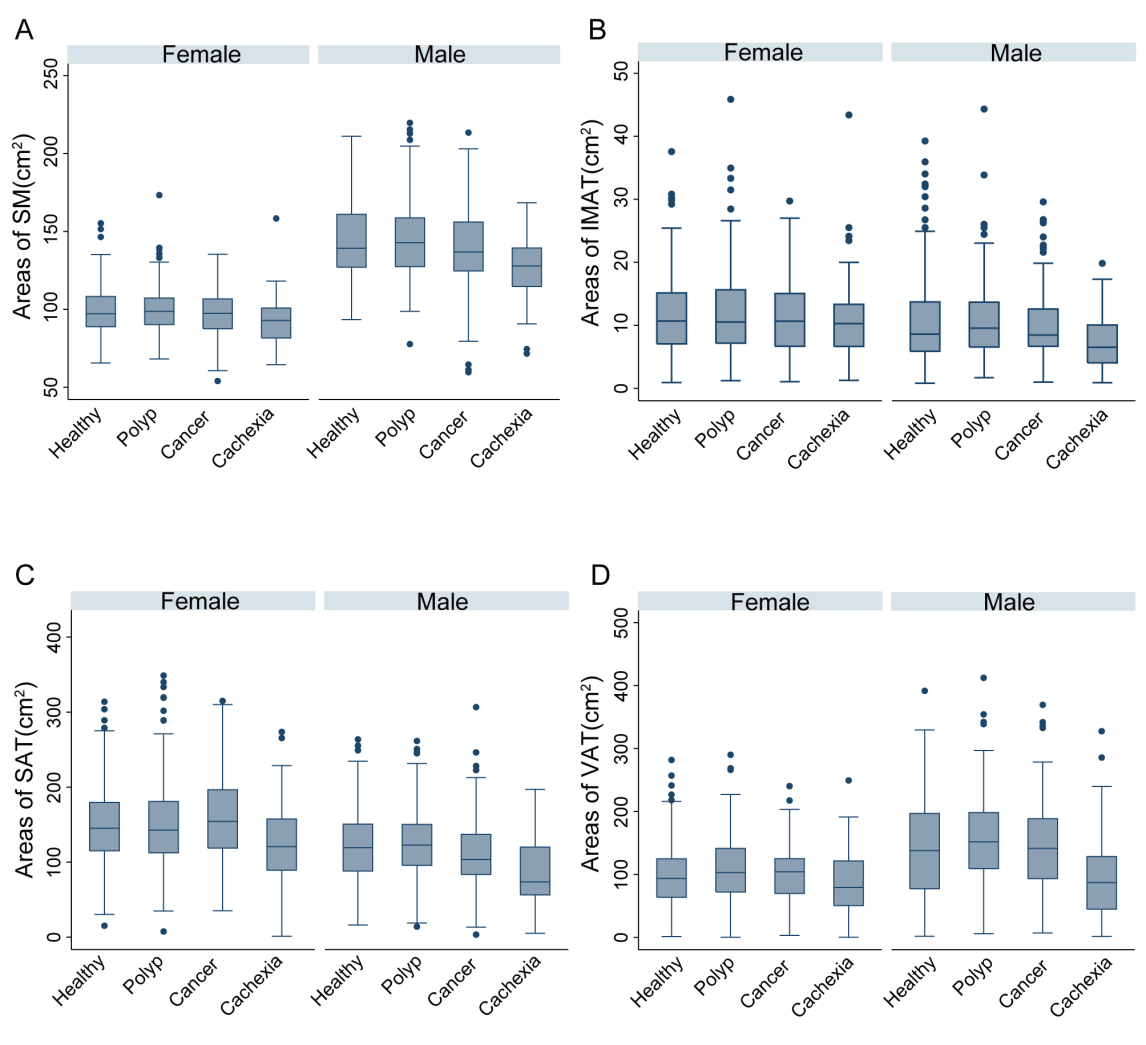
Comparison of abdominal fat and muscle composition of four groups (healthy controls, polyp group, cancer group, and cachexia group) according to different genders. A: Comparison of skeletal muscle (SM) areas in four groups. B: Comparison of intramuscular adipose tissue (IMAT) areas in four groups. C: Comparison of subcutaneous adipose tissue (SAT) areas in four groups. D: Comparison of visceral adipose tissue (VAT) areas in four groups. *: *P* < 0.05

In this study, we included 483, 503, 399, and 128 patients in the healthy controls, polyp group, cancer group, and cachexia group, respectively. Table 1 showed the characteristics of age, gender, and abdominal fat and muscle composition of patients in each group. Significant differences were detected in age, gender and abdominal fat and muscle composition among the 4 groups (*P* < 0.05). The overall distributions of the abdominal fat and muscle composition in different genders were shown in Fig. 1.

**Table 1:** Patients’ characteristics of four groups

	Healthy (n = 483)	Poloyp (n = 503)	Cancer (n = 399)	Cachexia (n = 128)	*P*
Age	60.67 ± 11.62	62.82 ± 10.59	63.03 ± 10.53	63.29 ± 11.11	0.002
Male (%)	181(37.47)	267(53.08)	259(65.24)	67(54.03)	< 0.001
SM	115.87 ± 27.64	122.90 ± 30.00	125.21 ± 30.41	110.64 ± 25.81	< 0.001
IMAT	11.31 ± 6.58	11.44 ± 6.60	10.39 ± 5.62	9.38 ± 6.42	0.006
VAT	113.52 ± 64.95	133.61 ± 67.00	129.15 ± 69.23	90.80 ± 61.21	< 0.001
SAT	138.50 ± 53.40	138.68 ± 55.29	126.93 ± 54.84	103.96 ± 55.66	< 0.001

Note: SAT: subcutaneous adipose tissue, VAT: visceral adipose tissue. IMAT: intramuscular adipose tissue; SM: skeletal muscle

### Changes of abdominal fat and muscle composition from normal mucosa to polyp and cancer

**Fig. 2:**
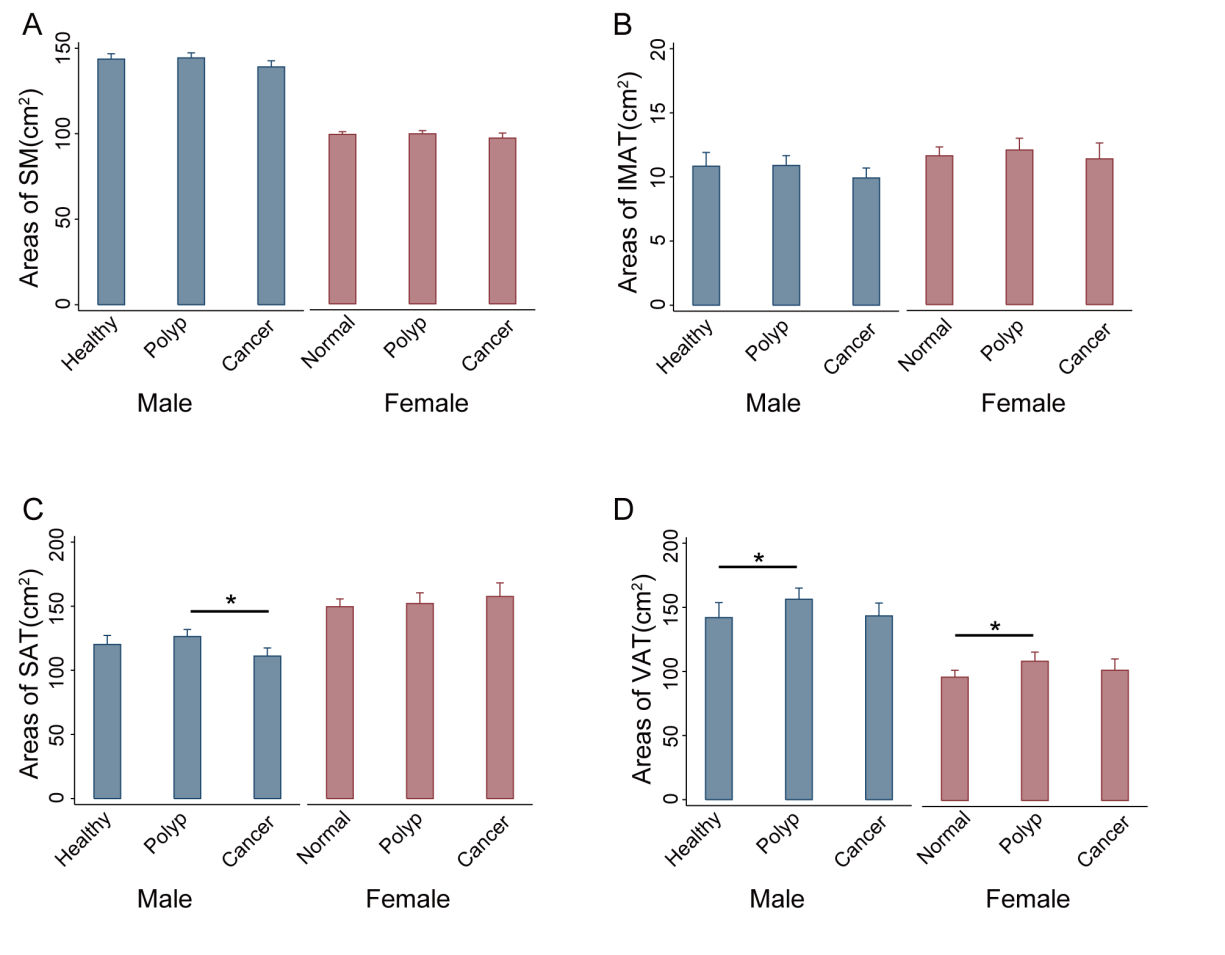
Abdominal fat and muscle composition changes in the process of CRC from normal mucosa to polyp and cancer according to different gender. A: Comparison of skeletal muscle (SM) areas in three groups (healthy controls, polyp group, cancer group). B: Comparison of intramuscular adipose tissue (IMAT) areas in three groups. C: Comparison of subcutaneous adipose tissue (SAT) areas in three groups. D: Comparison of visceral adipose tissue (VAT) areas in three groups. *: *P* < 0.05

Firstly, we compared the change of abdominal fat and muscle composition in the process of CRC from normal mucosa to polyp and cancer. Due to the obvious difference in abdominal fat and muscle composition between different genders, we compared the changes of each index according to different gender separately. As shown in Fig. 2, we found that there was no significant difference between the SM area and IMAT area among the healthy controls, polyp group, and cancer group in both genders. However, the VAT area of the polyp group was significantly higher than that of the healthy controls both in male (156.32 ± 69.71 cm^2^ vs. 141.97 ± 79.40 cm^2^, *P* = 0.014) and female patients (108.69 ± 53.95 cm^2^ vs. 96.28 ± 46.70 cm^2^, *P* = 0.044). There was no significant difference in VAT area both between the polyp group and cancer group, and between healthy controls and cancer group. There was no significant difference of SAT area between the polyp group and the healthy controls in male (126.40 ± 43.52 cm^2^ vs. 120.15 ± 48.47 cm^2^, *P* = 0.499) and female patients (152.16 ± 63.23 cm^2^ vs. 149.62 ± 53.25 cm^2^, *P* = 1.000). However, SAT area decreased significantly in the male cancer group compared with the polyp group (111.16 ± 46.98 cm^2^ vs. 126.40 ± 43.52 cm^2^, *P* = 0.001), while no such change was observed in female patients (157.62 ± 56.21 cm^2^ vs. 152.16 ± 63.24 cm^2^, *P* = 1.000). These results indicated that SAT begin to lose in male patients after the occurrence of CRC. The *P* values of these three groups compared with each other were shown in Table 2.

**Table 2:** *P* values of healthy group, polyp group, and cancer group compared with each other by different genders

	SM		IMAT		SAT		VAT	
	M	F	M	F	M	F	M	F
*P1*	1.000	1.000	1.000	1.000	0.499	1.000	0.044	0.014
*P2*	0.201	0.556	0.498	1.000	0.163	0.641	1.000	1.000
*P3*	0.065	0.416	0.335	1.000	0.001	1.000	0.180	0.647

Note: *P1*: polyp group VS. healthy group; *P2*: cancer group VS. healthy group; *P3*: cancer group VS. polyp group; SAT: subcutaneous adipose tissue, VAT: visceral adipose tissue. IMAT: intramuscular adipose tissue; SM: skeletal muscle; M: male; F: female

### Abdominal fat and muscle composition difference between different stages of CRC and healthy controls

To clarify the abdominal fat and muscle composition characteristics in different stages of CRC, we compared abdominal fat and muscle composition difference between healthy controls and 3 other groups. As shown in Table 3, abdominal fat and muscle composition was compared after adjustment for age and gender. The SM area of the cachexia group was significantly lower than healthy controls, with a mean reduction area of 9.25 cm^2^ (95% CI: 5.39–13.11 cm^2^, *P* < 0.001). There was no significant difference of SM area between polyp group and cancer group compared with the healthy controls. Similarly, we found that IMAT area was significantly lower only in the cachexia group, with a decreased area of 1.93 cm^2^ (95% CI: 0.54–3.32 cm^2^, *P* = 0.006). The SAT area was found slightly higher in the polyp group and lower in the cancer group, and decreased significantly in the cachexia group, with a reduction area of 28.84 cm^2^ (95% CI: 17.84–39.83 cm^2^, *P* < 0.001). Interestingly, we found that the VAT area of the polyp group increased significantly by 12.19 cm^2^ (95% CI: 4.18–20.20 cm^2^, *P* = 0.003) compared to the healthy controls. There was no significant difference of VAT area between the cancer group and the healthy controls, while the VAT area of the cachexia group decreased significantly by 31.31 cm^2^ (95% CI: 18.12–44.51 cm^2^, *P* < 0.001) compared to the healthy controls.

**Table 3:** Relative changes of abdominal fat and muscle composition in different stages of CRC compared to healthy group

	SM				IMAT				SAT				VAT			
	Univariate		Multivariate		Univariate		Multivariate		Univariate		Multivariate		Univariate		Multivariate	
	β	*P*	β	*P*	β	*P*	β	*P*	β	*P*	β	*P*	β	*P*	β	P
Age	-0.73 (-0.86, -0.59)	< 0.001	-0.74 (-0.83, -0.65)	< 0.001	0.20 (0.17, -0.23)	< 0.001	0.20 (0.17, 0.23)	< 0.001	-0.40 (-0.66, -0.14)	0.003	-0.37 (-0.62, -0.12)	0.004	0.92 (0.60, 1.23)	< 0.001	0.91 (0.61, 1.21)	< 0.001
Sex	42.77 (40.64, 44.90)	< 0.001	43.15 (41.17, 45.12)	< 0.001	-1.35 (-2.04, -0.67)	< 0.001	-1.21 (-1.87, -0.56)	< 0.001	-33.38 (-38.95, -27.81)	< 0.001	-33.01 (-38.62, -27.39)	< 0.001	43.16 (36.41, 49.91)	< 0.001	42.63 (35.89, 49.36)	< 0.001
Healthy	0.00	---	0.00	---	0.00	---	0.00	---	0.00	---	0.00	---	0.00	---	0.00	---
Polyp	7.03 (3.35, 10.71)	< 0.001	2.11 (-0.21, 4.44)	0.074	0.14 (-0.67, 0.95)	0.740	-0.08 (-0.85, 0.68)	0.833	0.18 (-6.75, 7.11)	0.959	5.50 (-1.18, 12.17)	0.106	20.09 (11.65, 28.53)	< 0.001	12.19 (4.18, 20.20)	0.003
Cancer	9.33 (5.17, 13,49)	< 0.001	-1.48 (-4.15, 1.19)	0.277	-0.92 (-1.86, 0.02)	0.055	-1.06 (-1.96, -0.16)	0.021	-11.57 (-19.27, -3.86)	0.003	-1.29 (-8.83, 6.24)	0.736	15.62 (6.25, 25.00)	0.001	1.26 (-7.78, 10.30)	0.784
Cachexia	-5.23 (-11.39, 0.92)	0.095	-9.25 (-13.11, -5.39)	< 0.001	-1.93 (-3.32, -0.54)	0.006	-2.29 (-3.59, -0.99)	0.001	-34.54 (-46.04, -23.05)	0.001	-28.84 (-39.83, -17.84)	< 0.001	-22.72 (-36.71, -8.73)	0.001	-31.31, (-44.51, -18.12)	< 0.001

Note: β(cm^2^); SAT: subcutaneous adipose tissue, VAT: visceral adipose tissue. IMAT: intramuscular adipose tissue; SM: skeletal muscle; M: male; F: female

### Relative changes of abdominal fat and muscle composition in cachexia group compared to other groups

We focused on comparing the changes of abdominal fat and muscle composition in CRC patients with cachexia. By comparing the abdominal fat and muscle composition between the cachexia group and 3 other groups, we found that there were significant differences between the cachexia group and any other groups (Table 4). As shown in Fig. 1, significant difference was detected between cachexia group and other groups, regardless of male and female patients. Most of all, the SAT area of cachexia group deceased 28.84 cm^2^ (95% CI: 17.84–39.83 cm^2^, *P* < 0.001), 34.33 cm^2^ (95% CI: 23.38–45.27 cm^2^, *P* < 0.001), and 27.54 cm^2^ (95% CI: 16.11–38.98 cm^2^, *P* < 0.001), compared with the healthy controls, polyp group, and cancer group, respectively. The VAT area of cachexia group deceased 31.31 cm^2^ (95% CI: 18.12–44.51 cm^2^, *P* < 0.001), 43.51 cm^2^ (95% CI: 30.36–56.65 cm^2^, *P* < 0.001) and 32.57 cm^2^ (95% CI: 18.85–46.29 cm^2^, *P* < 0.001), compared with the healthy controls, polyp group, and cancer group, respectively. A higher extent of loss in VAT was observed in the cachexia group compared with SAT, suggesting that VAT was more metabolically active than SAT in CRC patients with cachexia.

**Table 4:** Relative changes of abdominal fat and muscle composition in CRC patients with cachexia compared to other groups

	SM				IMAT				SAT				VAT			
	Univariate		Multivariate		Univariate		Multivariate		Univariate		Multivariate		Univariate		Multivariate	
	β	P	β	P	β	P	β	P	β	P	β	P	β	P	β	P
Age	-0.73 (-0.86, -0.59)	< 0.001	-0.74 (-0.83, -0.65)	< 0.001	0.20 (0.17, -0.23)	< 0.001	0.20 (0.17, 0.23)	< 0.001	-0.40 (-0.66, -0.14)	0.003	-0.37 (-0.62, -0.12)	0.004	0.92 (0.60, 1.23)	< 0.001	0.91 (0.61, 1.21)	< 0.001
Sex	42.77 (40.64, 44.90)	< 0.001	43.15 (41.17, 45.12)	< 0.001	-1.35 (-2.04, -0.67)	< 0.001	-1.21 (-1.87, -0.56)	< 0.001	-33.38 (-38.95, -27.81)	< 0.001	-33.01 (-38.62, -27.39)	< 0.001	43.16 (36.41, 49.91)	< 0.001	42.63 (35.89, 49.36)	< 0.001
Healthy	5.23 (-0.92, 11.39)	0.095	9.25 (5.39, 13.11)	< 0.001	1.93 (0.54, 3.32)	0.006	2.29 (0.99, 3.59)	0.001	34.54 (23.05, 46.04)	< 0.001	28.84 (17.84, 39.83)	< 0.001	22.72 (8.73, 36.71)	0.001	31.31 (18.12, 44.51)	< 0.001
Polyp	12.24 (6.10, 18.41)	< 0.001	11.36 (7.52, 15.21)	< 0.001	2.07 (0.68, 3.46)	0.004	2.21 (0.91, 3.50)	0.001	34.72 (23.23, 46.22)	< 0.001	34.33 (23.38, 45.27)	< 0.001	42.81 (28.82, 56.80)	< 0.001	43.51 (30.36, 56.65)	< 0.001
Cancer	14.57 (8.12, 21,09)	< 0.001	7.76 (3.72, 11.80)	< 0.001	1.01 (-0.46, 2.48)	0.180	1.22 (-0.15, 2.60)	0.080	22.97 (11.00, 34.95)	< 0.001	27.54 (16.11, 38.98)	< 0.001	38.35 (23.77, 52.92)	< 0.001	32.57 (18.85, 46.29)	< 0.001
Cachexia	0.00	---	0.00	---	0.00	---	0.00	---	0.00	---	0.00	---	0.00	---	0.00	---

Note: β(cm^2^); SAT: subcutaneous adipose tissue, VAT: visceral adipose tissue. IMAT: intramuscular adipose tissue; SM: skeletal muscle; M: male; F: female

## Discussion

In this study, by comparing the abdominal fat and muscle composition in patients with and without colorectal polyp, CRC patients with and without cachexia, we found significant differences of abdominal fat and muscle composition in different stages of CRC. Most importantly, VAT area was the largest in patients with colorectal polyp compared to other groups. In CRC patients with cachexia, the areas of SAT, VAT, SM, as well as IMAT were all found decreased significantly compared to other groups. This is the first comprehensive study focused on the of abdominal fat and muscle composition difference during the different stages of CRC progression from normal mucosa to polyp to cancer and cachexia.

Although the association between VAT and CRC was controversial, its association with colorectal polyp was quite well established [31, 32]. Various studies have demonstrated that increase in VAT area was an independent risk factor for colorectal polyp [33]. In this study, significant increase of VAT area was found in the patients with polyp compared with the healthy controls both in male and in female. Further research showed that the increase of VAT in female was higher than that in male, suggesting that the increase of VAT was more likely to promote the occurrence of adenoma in female patients. No significant increase of SAT area was observed in patients with polyp in both genders, which was consistent with the previous study [34]. These results suggested that the growth of VAT but not SAT was a risk factor for colorectal polyp, and female patients should pay more attention to visceral obesity.

VAT related inflammation was supposed to promote CRC initiation and progression [10]. However, Akay et al. reported that areas of VAT and SAT decreased in CRC patients compared with the healthy controls [35]. In this study, we found that the VAT area in CRC patients without cachexia was slightly higher than that of healthy controls, while slightly lower than that of patients with colorectal polyp. This suggested that the VAT was the largest area in patients with colorectal polyp, and VAT began to decrease when cancer occurs. We also found that the SAT area in CRC patient without cachexia was significantly lower than patients with polyp in male, while slightly higher than patients with polyp in female. These results suggests that SAT and VAT may have different roles during the development of CRC.

According to the consensus of cancer cachexia proposed in 2011, patients with cancer cachexia were characterized by muscle loss with or without adipose tissue loss [25]. However, a large number of studies have found that most of cancer patients with cachexia such as gastric cancer and pancreatic cancer were associated with adipose tissue loss [36]. As commonly known, obese patients are more likely to suffer from CRC. When cancer cachexia develops, it is unclear whether adipose tissue will also be significantly decreased on the basis of obesity. In this study, we found both areas of SAT and VAT in patients with cachexia were significantly lower than those of normal patents, polyp patients, and non-cachexia patients. This suggested adipose tissue loss was one of the important characteristics of CRC patients with cachexia.

Ebadi et al. reported that loss of VAT precedes SAT in advanced colorectal and cholangiocarcinoma cancer patients [37]. However, SAT, but not VAT, began to lose in male patients without cachexia in this study, while the loss degree of VAT is greater than that of SAT. Although the underlying mechanism is still not very clear, VAT contains more immune cells than SAT, which may promote lipolysis [8]. More attention should be paid to the SAT loss in the early stage of CRC and VAT loss in the late stage of CRC.

As one of the most important characteristics of cancer cachexia, the study of SM atrophy and its mechanism was more comprehensive than that of adipose tissue loss [38]. In this study, we compared the difference of SM area in different stages of CRC. There was no significant change of SM area among the healthy controls, polyp group, and cancer group, suggesting that SM atrophy did not exist until the early stage of CRC. SM area began to decrease in patient with cachexia, demonstrating that adipose tissue loss precedes muscle loss in CRC patient. It was also suggested that cancer cachexia might be caused by the interaction of muscle and adipose tissue [39].

To our knowledge, this is the first analysis to investigate the IMAT area in different stages of CRC. Interestingly, we found that the IMAT area was smaller in cachexia patients than that in healthy controls and polyp patient, while was similar with CRC patient without cachexia. Nevertheless, as IMAT is a novel topic in adipose tissue depot, more data is needed to validate our findings.

Our study has several limitations. First, this is a retrospective cross-sectional study and has a relatively small cachexia sample size. Second, IMAT area varies greatly among different groups, the final difference in different groups still need to be determined by further research. Thirdly, this study mainly includes two major confounding factors: age and gender. It is unclear whether there are other factors that affect the results. Despite these limitations, we still believe that subcutaneous and visceral adipose tissue play different roles in the different stages of CRC development.

## Conclusion

Our research provides important insights into the abdominal fat and muscle composition, especially subcutaneous and visceral adipose tissue, in different stages of colorectal cancer. These findings provide a novel understanding of the association between adipose tissues and CRC. Further studies are essential to understand how different part of adipose tissues, such as SAT, VAT and IMAT, affect CRC progression.

## Electronic supplementary material

Below is the link to the electronic supplementary material.

Supplementary Material 1

## Data Availability

The raw data supporting the conclusions of this article will be made available by the corresponding author Guohao Wu, upon reasonable request.

## Abbreviations

CRC: colorectal cancer
SAT: subcutaneous adipose tissue
VAT: visceral adipose tissue
IMAT: intramuscular adipose tissue
SM: skeletal muscle
CT: computed tomography
