# Supplementary material for: Abdominal fat and muscle distributions in different stages of colorectal cancer
Source: BMC Cancer. 2023 Mar 28;23:279. doi: 10.1186/s12885-023-10736-2 (PMC10044362; doi:10.1186/s12885-023-10736-2)
Supplement: Supplementary file 1 — Supplementary Material 1 [file 12885_2023_10736_MOESM1_ESM.docx]

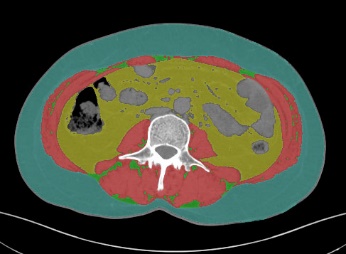


**Fig. S1.** A representative CT image at the third lumbar vertebra (L3) marked with different abdominal fat and muscle composition. Skeletal muscle (SM) in red; subcutaneous adipose tissue (SAT) in blue; visceral adipose tissue (VAT) in yellow; intramuscular adipose tissue (IMAT) in green.
